# Supplementary material for: MetaRibo-Seq measures translation in microbiomes
Source: Nat Commun. 2020 Jun 29;11:3268. doi: 10.1038/s41467-020-17081-z (PMC7324362; doi:10.1038/s41467-020-17081-z)
Supplement: Supplementary file 10 — Supplementary Data 7 [file 41467_2020_17081_MOESM10_ESM.zip › File2/Confidence_High_Taxonomy/402953_out.krona.html]

Javascript must be enabled to view this page.

members
magnitude
magnitudeUnassigned
count
unassigned
taxon
rank

402953\_out

15

15
2
superkingdom

15
976
phylum

class
200643
15

15
order
171549

1
815
family

1
genus
816

species
28116

SRS104327\_contig\_number\_73
1

family
171552
14

14
genus
838


SRS103987\_contig\_number\_1652
1
species
59823


SRS013098\_contig\_number\_38082SRS014415\_contig\_number\_1815SRS018888\_contig\_number\_2973SRS019910\_contig\_number\_23386SRS024132\_contig\_number\_contig-100\_569.291768SRS046502\_contig\_number\_13643SRS047433\_contig\_number\_15726SRS074586\_contig\_number\_5175SRS077194\_contig\_number\_11016SRS104036\_contig\_number\_6562SRS1041137\_contig\_number\_22403SRS144506\_contig\_number\_45745SRS150029\_contig\_number\_17887
13
species
165179
